# The Role of Inclusion Binding Contributions for β-Cyclodextrin Polymers Cross-Linked with Divinyl Sulfone?—A Comment on Morales-Sanfrutos *et al.* Entitled “Divinyl Sulfone Cross-Linked Cyclodextrin-Based Polymeric Materials: Synthesis and Applications as Sorbents and Encapsulating Agents”, *Molecules*, 2015, *20*, 3565–3581.

**DOI:** 10.3390/molecules21010093

**Published:** 2016-01-14

**Authors:** Lee D. Wilson, Mohamed H. Mohamed, Dena W. McMartin

**Affiliations:** 1 Department of Chemistry, College of Arts and Science, University of Saskatchewan, 110 Science Place, Saskatoon, SK S7N 5C9, Canada; mom133@mail.usask.ca; 2 Environmental Systems Engineering, Faculty of Engineering and Applied Science, University of Regina, 3737 Wascana Parkway, Regina, SK S4S0A2, Canada; Dena.McMartin@uregina.ca

**Keywords:** β-cyclodextrin, cross-linking, divinyl sulfone, adsorption, inclusion binding, polymer, phenolphthalein

## Abstract

This commentary reports on a recent scientific study reported in this journal (*cf*. *Molecules* **2015**, *20*(3), 3565–3581). Some key scientific issues that require further explanation and clarification in the former article are as follows: (i) the relationship between the inclusion site accessibility and the level of cross-linking employed are brought into question for the case of α-CD and β-CD cross-linked adsorbent materials; (ii) the binding affinity of the CD/guest complexes were not related to the isotherm parameters for the CD-polymer/guest systems; (iii) the limited molecular level structural characterization of the cross-linked polymer materials; and (iv) the interpretation of the adsorption isotherm results by the authors.

We wish to offer some comments on the article by Morales-Sanfrutos *et al.* entitled “Divinyl Sulfone Cross-Linked Cyclodextrin-Based Polymeric Materials: Synthesis and Applications as Sorbents and Encapsulating Agents”, published in *Molecules* in 2015 [1]. In the latter report, the authors cross-linked a series of polysaccharides (α-CD, β-CD, and starch) using divinyl sulfone (DVS) at variable composition to form a series of insoluble homo- and hetero-polymers. There are several key scientific issues that require further explanation and clarification in the title article [1] by the authors:

1. The relatively high content of DVS in the preparation of the polymers is questioned (cyclodextrin (CD): DVS mole ratio (1:3.5; 1:7; and 1:14)) since the relationship between the inclusion site accessibility is known to decrease dramatically in the case of α-CD and β-CD cross-linked adsorbent materials.
2. The role of the binding affinity of the CD/guest complex was not related to the isotherm parameters for the βCD-P1/guest and αCD-P1/guest systems (phenol, *p*-nitrophenol, bisphenol, 2-naphthol, curcumin, and progesterone). The authors have unequivocally stated that “*cross-linking* *does not influence the extent of inclusion complex formation*”.
3. The paper presents very limited molecular level structural characterization of the cross-linked polymer materials and there remains some uncertainty about the level of cross-linking according to the CD: DVS ratios employed.
4. The reliability of linearized adsorption models are questioned on the basis of statistical bias of data at low concentration and the potential “masking” of real data trends. As well, it is difficult for the reader to visually assess the isotherm profile, in contrast to the presentation of isotherms in a conventional nonlinear manner (*Q_e_* *vs.* *C_e_*). The inadvertent use of excessive amounts of organic co-solvent may introduce artefacts into the design of adsorption experiments since such co-solvents are known to influence hydrophobic driven forces such as the formation of cyclodextrin inclusion complexes.

Previous studies have established the utility of a dye adsorption method that employs phenolphthalein (phth) to evaluate the inclusion site accessibility of cross-linked polymers containing β-CD [2,3]. A key feature of this method is the formation of a stable inclusion complex between β-CD and phth, along with unique changes for the photophysical properties of phth, where phth becomes optically transparent in the bound state (β-CD/phth), while it absorbs strongly in the visible region (λ = 552 nm) in its unbound state. The details of the dye adsorption method for the study of insoluble polymers containing β-CD have been described [2,3]. Studies of the inclusion site accessibility for cross-linked polymers containing β-CD reveal that steric effects exist at relatively low levels of cross-linking [2]. In the case of β-CD cross-linked with diisocyanates, significant attenuation of the inclusion site accessibility was observed for β-CD: diisocyanate mole ratios from 1:1 to 1:3 (*cf*. Table 2 in [2]). Steric effects related to the formation of inclusion complexes were demonstrated in a follow-up study [3] over a wider range of cross-linker types and mole ratios (*cf*. Table 1 in [3]).

To establish whether similar trends apply in the case of cross-linked DVS polymers containing β-CD, we repeated the synthesis of β-CD homopolymers reported by Morales-Sanfrutos *et al.* [1], including some cross-linking ratios at much lower levels not reported in their study, as described in Table 1 below. The heteropolymers containing starch were not examined since such materials have greater structural variability than β-CD and less sensitive to the phth dye-based method [2]. It should be noted that we define the mole ratio of cross-linking as β-CD: DVS to compare with an independent study reported previously (*cf*. Table 2 in [2]). According to Morales-Sanfrutos *et al.*, the β-CD: DVS polymers were deemed to have optimal adsorption properties (attributed to inclusion complex formation). The DVS content was expressed relative to the glucose content (n) of each respective polysaccharide (β-CD; *n* = 7) in their study [1]. The authors report that no difference in the inclusion efficacy was noted for higher *vs.* lower levels of DVS for β-CD: DVS systems (1:3.5; 1:7; and 1:14) [1]. We disagree with the following statement, “*In general*, *the degree of cross-linking does not seem to play a significant influence on the properties of the material*” [1]. The disagreement is based on steric effects described above for related polymers [2] along with the results presented from our study in Table 1.

**Table 1:** Inclusion site accessibility of β-CD homopolymers cross-linked with DVS.

Materials ^1^	Accessibility of β-CD Sites (%) ^2^
β-CD:DVS-(1:1)	47.9 (2.4)
β-CD:DVS-(1:2)	27.5 (1.4)
β-CD:DVS-(1:3) *	9.55 (0.50)
β-CD:DVS-(1:6) *	1.37 (0.070)

^1^ The synthetic feed ratio defined above relates to the mole ratio of β-CD to DVS (not according to the glucose monomer to DVS ratio as in reference [1]); ^2^ According to a standard error of 5% denoted in parentheses.* Denotes homopolymers that resemble the polymer composition reported in ref. [1].

Table 1 lists four types of cross-linked polymers (β-CD: DVS; 1:1 to 1:6) that were evaluated according to their inclusion site accessibility using the phth dye adsorption method [2]. The materials were synthesized according to the verbatim reported method [1]; however, the DVS content covers a wider range of values, particularly at lower levels of DVS content. The results for sulfur content are not reported in lieu of the verbatim synthetic procedure [1] followed for the β-CD: DVS homopolymers. The accompanying results for the inclusion site accessibility are listed below in Table 1.

In Table 1, the variable inclusion site accessibility reinforces the important role of steric effects, even at low levels of cross-linking, according to the synthetic feed ratios (1:1 to 1:3) in Table 1 above. Note that the lowest DVS content examined by Morales-Sanfrutos *et al.* correspond to 1:3.5 β-CD: DVS mole ratio. Thus, it should be noted that the polymers investigated in their study were focused on materials with higher levels of DVS overall, as evidenced by the synthetic ratios in their study (β-CD: DVS; 1:7 and 1:14). According to the results in Table 1, the inclusion site accessibility of phth with the cross-linked polymer (1:6) is relatively low (*ca*. 1.4%), further supporting the need for a re-interpretation of the work presented by Morales-Sanfrutos *et al.* [1]. The foregoing is especially relevant to the study of larger adsorbates such as bisphenol, 2-naphthol, curcumin, and progesterone, including a companion study by the same authors [4] in the journal *Molecules*. The phenolic and bioactive compounds listed above are relevant to the accessibility results in Table 1 due to their comparable molecular size with phth. Hence, the reported adsorption results [1] are likely due to attenuated formation of inclusion complexes due to steric effects described above, in contrast to the opposite conclusions reported by Morales-Sanfrutos *et al.* The authors conclude that no apparent difference in the formation of inclusion complexes was noted for the following homopolymers: β-CD: DVS systems prepared at the 1:14, 1:7, and 1:3.5 mole ratios [1]. The conclusions reported by Morales-Sanfrutos *et al.* [1] are in direct contrast with the results listed in Table 1.

The occurrence of dual mode adsorption (inclusion and non-inclusion) for CD-based polymers was suggested in a previous adsorption study of phenolic compounds by Garcia-Zubiri and coworkers [5] in 2009. They proposed an equilibrium model that accounts for inclusion complex formation by a hole-filling mechanism, based on the adsorption properties of CD cross-linked polymers (*cf*. Equation (5) in reference [5]) with phenol and 1-naphthol adsorbates. A recent isotherm study further elaborates on the role of inclusion *vs.* non-inclusion sites and the adsorption properties for related CD-based polymers [6]. The foregoing results in Table 1, along with the literature precedence for dual mode adsorption, further indicates that the title study [1] has largely neglected the importance of contributions due to non-inclusion binding, along with a related adsorption study by the same research group in the same issue of this journal [4]. The interpretation that inclusion binding is the sole contributor to the adsorption process for CD-based polymers highlights the origin of the inconsistency, as evidenced by the isotherm results in Tables 3–5 for the homopolymers in reference [1]. The lack of correspondence of the isotherm parameters with the reported binding affinities for the inclusion complexes of such β-CD/guest systems in Tables 3–5 are likely due to an improper account of dual mode adsorption, especially contributions arising from non-inclusion sites (cross-linker domains) within the polymer network [5]. Further evidence of such discrepancies is evidenced in [1] by the use of the Langmuir *vs.* Freundlich isotherms for various cross-linked polysaccharide systems, since each model accounts for homogeneous *vs.* heterogeneous adsorption properties, respectively. The foregoing is in accordance with the likely role of dual mode binding that has not been adequately accounted for in reference [1], as described above. The authors concluded that the formation of inclusion complexes was the key contribution for the adsorption of guests with variable size; however, it should be noted that cross-linked starch (S-P1) has notable adsorption toward 2-napthol and progesterone (*cf.* Tables 4 and 5 in reference [1]). The adsorption properties of S-P1 provide indirect evidence that non-inclusion binding is important since starch does not possess a pre-organized macrocyclic binding site, as compared with β-CD and its homopolymers.

As mentioned in query 3 and 4 above, the limited molecular level characterization of the homopolymers, along with the use of linearized fitting of the isotherm results, leads to an improper assessment of the role of non-inclusion contributions to adsorption, according to the dual mode adsorption model [5]. The role of inclusion binding is further exacerbated in the title study [1] for the adsorption of curcumin and progesterone. This is due to the use of organic co-solvents (10% *w*/*w*) since the use of such high levels of co-solvent are known to attenuate hydrophobic driven processes, especially in the case of the formation of cyclodextrin inclusion complexes. Significant amounts of co-solvent may further attenuate the formation of inclusion complexes [7] since such mixtures adulterate the properties of an aqueous environment, as for the case of neat water. The concluding remarks made by the authors that the “*cross-linking exerts a clear influence on the surface of the material*, *whereas it plays a minor role on the formation of the inclusion complexes*” is contradictory given the likely role of dual mode adsorption.

We thus appeal to authors to promptly clarify the scientific issues outlined above and to provide further clarification on reported discrepancies.
